# Acidosis in Children

**Published:** 1913-07-19

**Authors:** C. Willett Cunnington


### ACIDOSIS IN CHILDREN.

By C. WILLETT CUNNINGTON, M.B., B.C.

THIS condition has attracted a good deal of attention in recent years. It should prove of special interest to those in general practice, for it will be found, if routine investigations are made, that moderate degrees of it are frequently occurring, and are overlooked because the urine is not fully examined. Severe cases are not uncommon, and remain undiagnosed for the same reason.

In that important group of diseases which are responsible for a clinical condition of " fever without physical signs " a place must be reserved for acidosis. Whatever the actual pathology of the condition may be, we may briefly describe it as characterised by the presence of acetone and allied bodies (diacetic and $\beta$-oxybutyric acids) in the urine. Diacetic acid is revealed by the well-known ferric chloride test, but Rothera's test for acetone is more sensitive, and is in itself, therefore, sufficient. It has been recently suggested that in reality this reaction depends on the presence of diacetic acid, and not acetone. The two substances are so closely allied, however, that for clinical purposes the distinction does not matter.

Rothera's test consists in adding to the urine an equal bulk of saturated solution of ammon. sulphate, followed by liq. ammoniæ, and then a few drops of 5-per-cent. solution of sodium nitroprusside. A purple colour, appearing either at once or shortly, indicates the presence of acetone.

Acetonuria in diabetes is familiar enough as a herald of coma, and in the condition known as "delayed chloroform poisoning "; but it is desirable to recognise that there are also much commoner conditions in childhood, in which acetonuria may be present. Naturally, it is the general practitioner who is likely to meet with such.

Certain children are prone to suffer from what is likely to be called " bilious attacks." In my own experience I have found a typical attack to be preceded by a few days of lassitude, with constipation and a furred tongue. The temperature then rises, sometimes only a degree or so, but not seldom to 102°. The pyrexia lasts several days, during which the child is drowsy and declines food. Occasionally the bowels are relaxed and the stools offensive. Often headache is the chief symptom complained of. In a word, we have a condition of " fever without physical signs," and it may last long enough to suggest enteric fever. There is no leucocytosis. The child usually feels sick, and may actually vomit.

One distinguishes such attacks from the condition described by Gee as " periodic vomiting " on clinical grounds, but perhaps the distinction is only one of degree. In the former condition there may be some abdominal pain or discomfort, worse if retching is a feature. In such cases a hasty diagnosis of mild " appendicitis " is a mistake easily made. The diagnosis is made by finding acetone in the urine, and this, at the beginning of the attack, not merely the result of vomiting or starvation.

The more severe condition of " periodic or cyclical vomiting " is much rarer, and appears to be more dangerous. Vomiting is constant and continuous, even though the patient is receiving nothing by mouth. The temperature is not raised; often it is subnormal. Physical exhaustion is easily and rapidly produced, and may give rise to considerable anxiety. In a case seen recently the pulse rate rose in the course of a day to 140, small and irregular.

Cyclical vomiting seldom begins under the age of five, and ceases spontaneously at puberty. The attacks recur at intervals of a few months. The children subject to it are usually of the thin, "nervous" type, somewhat anæmic, and "finicky" in their appetite. Inasmuch as it seems reasonable to regard the condition as being in the nature of a toxæmia due to faulty metabolism of fats and carbohydrates, the toxins accumulating in the liver (perhaps), until eventually an " explosion " occurs (suggesting an analogy to gout), we should expect these patients to have capricious digestions, and this is found to be the case.

The tendency to acidosis in certain children is an important clinical feature to appreciate, because it is becoming established that it is just these children who are peculiarly liable to " delayed chloroform poisoning " after anæsthesia. Such children, then, should not be operated upon without special precautions to diminish the risk to which they have this curious susceptibility. Fortunately, we are able to accomplish something in this direction.

It seems desirable to limit the amount of starchy foods, especially as we often find these children receive an excess of these substances; and in addition the fats are to be kept within bounds. At the first signs of an attack (heralded by drowsiness, furred tongue, and acetonuria) the fats should be withheld entirely. To neutralise the acidosis large quantities of alkali, such as lime water or sodium bicarbonate, are given, until the urine has become markedly alkaline.

In delayed chloroform poisoning it is beneficial to give large doses of an easily absorbed sugar; and, arguing by analogy, it seems reasonable to give the same to children showing the first signs of acidosis. Barley-sugar is both effective and popular.

Suppose the patient has already begun to vomit, it is worth while trying sodium bicarbonate and sugar by mouth, as sometimes these will be retained when nothing else will, but valuable time should not be lost if vomiting is frequent and exhaustion threatens. In such cases it is best to resort at once to proctolysis. By far the best method is the continuous rectal infusion, using some apparatus on the principle of the Thermos flask, whereby the liquid is kept at a constant temperature, and is given without intermission for twelve or twenty-four hours, or longer if need be. A child of seven will absorb six to eight ounces per hour. The fluid should enter the bowel at a temperature of 105°. This necessitates a higher temperature in the

Thermos flask (a point often forgotten by nurses). By previous experiment this is usually found to be about 120° to 130° F. The fluid may be normal saline, but better is a 5-per-cent. solution of glucose and sodium bicarbonate. If this is not retained it can be given, in a sterile form, under the skin. If vomiting continues, morphia hypodermically may be necessary.

Lastly, if a child, known to be subject to acidosis, requires any operation, the most suitable time to choose is soon after an attack, when the system has, as it were, "disgorged" its excess of metabolic poison. In any case, it is wise to give the patient liberal doses of barley-sugar and sodium bicarbonate the day before the operation, and to omit the conventional purge. One has to bear in mind that any condition associated with vomiting is likely to produce some acetonuria, with perhaps acetone in the breath. Such are not to be confused with true acidosis, which is distinguished by the presence of acetone at the very beginning of the attack.

